# Latent Effects of Hsp90 Mutants Revealed at Reduced Expression Levels

**DOI:** 10.1371/journal.pgen.1003600

**Published:** 2013-06-27

**Authors:** Li Jiang, Parul Mishra, Ryan T. Hietpas, Konstantin B. Zeldovich, Daniel N. A. Bolon

**Affiliations:** 1 Department of Biochemistry and Molecular Pharmacology, University of Massachusetts Medical School, Worcester, Massachusetts, United States of America; 2 Program in Bioinformatics and Integrative Biology, University of Massachusetts Medical School, Worcester, Massachusetts, United States of America; The University of Arizona, United States of America

## Abstract

In natural systems, selection acts on both protein sequence and expression level, but it is unclear how selection integrates over these two dimensions. We recently developed the EMPIRIC approach to systematically determine the fitness effects of all possible point mutants for important regions of essential genes in yeast. Here, we systematically investigated the fitness effects of point mutations in a putative substrate binding loop of yeast Hsp90 (Hsp82) over a broad range of expression strengths. Negative epistasis between reduced expression strength and amino acid substitutions was common, and the endogenous expression strength frequently obscured mutant defects. By analyzing fitness effects at varied expression strengths, we were able to uncover all mutant effects on function. The majority of mutants caused partial functional defects, consistent with this region of Hsp90 contributing to a mutation sensitive and critical process. These results demonstrate that important functional regions of proteins can tolerate mutational defects without experimentally observable impacts on fitness.

## Author Summary

Changes in protein sequence or expression strength can both lead to adaptation in natural systems. While many studies have focused individually on either expression strength or protein sequence, in principle the fitness effects of these two protein properties are interdependent. We systematically investigated the fitness effects of both expression strength and protein sequence for the yeast Hsp90 gene (Hsp82). We analyzed the fitness effects of all possible point mutations in a putative substrate binding loop under seven different expression strengths. The fitness effects of amino acid substitutions were strongly dependent on expression strength. Many point mutations exhibited fitness defects at reduced expression strength that were hidden at the natural expression strength. Revealing these hidden mutant defects suggested that this region of Hsp90 contributes to a rate-limiting step in function, consistent with its putative role in substrate binding. This study is important because it indicates that critical regions in proteins are more prevalent than would be estimated based on experimental fitness analyses performed at natural expression strengths. As hidden fitness effects are likely to occur in other systems, these findings have broad implications for the field of experimental evolution.

## Introduction

Genetic changes that alter protein sequence or expression level can lead to adaptation, suggesting these protein properties are central to evolutionary processes. Many studies have individually investigated the effects of changes to either protein sequence or expression level. For example, protein sequences have been optimized under selective pressure using in vitro evolution [1]. In addition, changes in protein sequence relative to synonymous substitutions are a hallmark of positive selection in natural populations [2], [3]. The influence of protein expression level on fitness has also been well documented [4]. For example, changes to the expression level of the Agouti protein (but not its sequence) have been shown to affect fitness in wild mice by modulating coat coloration [5]. In addition, experiments in E. coli demonstrate that expression from the lac operon is rapidly tuned for optimal growth over a wide range of lactose concentrations [6]. While most studies to date have focused individually on either expression level or protein sequence, in principle the fitness effects of these two protein properties are interdependent [7], [8]. Here, we systematically investigate selection on the sequence and expression level of yeast Hsp90 (Hsp82).

We recently developed an approach termed EMPIRIC [9], which is a genetic screen that provides fitness measurements of all possible amino acid substitutions in short regions of important genes in yeast. By sampling across the variety of different amino acid substitutions, EMPIRIC provides detailed information about the physical constraints on protein function. We previously reported a bimodal distribution of fitness effects (DFE) for an evolutionarily conserved region of the yeast Hsp90 gene[9], an essential chaperone required for the maturation of many kinases [10]–[12]. Bimodal DFEs, where most mutants have fitness effects close to either null or wild type (WT), appear common in nature as they have been observed in many other fitness studies [13]–[17].

Bi-modal DFEs are consistent with a recently proposed model where the impacts of mutations on protein stability have a dominant impact on fitness [18]. This model is founded on two concepts: positions that contribute directly to rate-limiting steps in protein function are rare; and the natively folded structure is required for function. Under these conditions, selection results in stably-folded proteins [19], [20], such that modestly destabilizing mutations can be tolerated without dramatic changes to the fraction of natively folded protein molecules and hence function. Because protein folding is cooperative there is a narrow range of stability where both the folded and unfolded state are highly populated, consistent with relatively few mutations having intermediate function. In this stability-dominated model, mutations to critical functional positions (e.g. catalytic sites in enzymes) destroy activity, but are presumed rare and so do not contribute greatly to the DFE. Of note, the prevalence of positions in proteins that directly contribute to rate-limiting steps in protein function and the fragileness of these positions to mutation have not been thoroughly investigated.

The effects of mutations on protein function can be investigated based on fitness effects; however, fitness effects need not correspond directly to functional effects. For example, many essential proteins can be dramatically reduced in net function (defined here as the product of expression level and function per molecule) without dramatic reductions of fitness [13], [21]–[25]. Heterozygotes with one null allele are often highly fit, indicating that 50% reductions in net function can be tolerated [26]. The relationship between fitness and the net function of a protein is formally an elasticity function [21]. Around the wild type net function, the elasticity function often has a slope less than one indicating that reductions in net function have dampened impacts on fitness [27], [28]. Experimental analyses of fitness effects are also constrained by experimental measurement precision, which is currently on the order of 1% [29]. In natural systems, the resolution of selection depends upon the inverse of effective population size and is on the order of 10^−7^ for yeast [30], [31]. Thus, the effects of mutations on function that are important in natural selection can be hidden to experimental fitness analyses. For example, the net function of lysozyme in phage T4 must be reduced about 30-fold before experimentally measurable impacts on growth are observed [13]. At the endogenous expression level in this system, large defects in per molecule function are hidden to experimental fitness analyses.

We searched for hidden fitness effects in Hsp90 by examining the Hsp90 elasticity function. We varied the expression level of the native protein sequence and monitored effects on yeast growth rate. Determining theHsp90 elasticity function enabled us to estimate mutant effects on per molecule function from fitness measurements. The elasticity function was non-linear such that at the endogenous expression level, mutant defects up to 79% in per molecule function were hidden to experimental fitness analyses. To reveal potentially hidden functional defects of mutants, we repeated EMPIRIC analyses at reduced expression strengths, which systematically varied fitness sensitivity to amino acid substitutions in Hsp90. Using this approach, we were able to construct a full distribution of mutant effects on function for a region of Hsp90. Structural analyses suggest that the region we chose to analyze is a putative substrate binding loop [32]. Our experimental fitness analyses at the wild type expression level resulted in a bimodal DFE, which is a hallmark of a scaffolding region with stability dominated effects on fitness [18]. By analyzing fitness at varied expression strengths, we found that the majority of Hsp90 point mutants had intermediate (10–90%) defects in per molecule function that were hidden to our analyses at wild type expression level. These observations indicate the region of Hsp90 we analyzed is involved in a rate-limiting step in function, and supports its putative role in binding to substrates [32].Because many mutant defects may be hidden to experimental measurement at the wild type expression level, our results suggest that rate-limiting functional sites in proteins may be more prevalent than previously appreciated, and provides a useful guide for interpreting the growing field of systematic mutant analyses [9], [33]–[40].

## Results/Discussion

While our initial EMPIRIC study [9] was performed with a temperature sensitive allele of Hsp90 co-expressed with all mutants; here, we report results in an Hsp90 shutoff strain where mutants were analyzed without potential co-expression artifacts. We developed a yeast shutoff strain (DBY288) where the only chromosomal copy of Hsp90 is regulated by a strictly galactose-dependent promoter [41]. In galactose media, the DBY288 strain expressed Hsp90 at endogenous levels and grew robustly. When switched to dextrose media, the DBY288 strain stalled in growth with Hsp90 levels rapidly dropping below detection (Supplementary Figure S1). This strain enabled plasmid encoded Hsp90 variants to be maintained and amplified under non-selective conditions (galactose media). Switching to dextrose media then applied selective pressure on the plasmid encoded Hsp90 variants.

**Figure 1:**
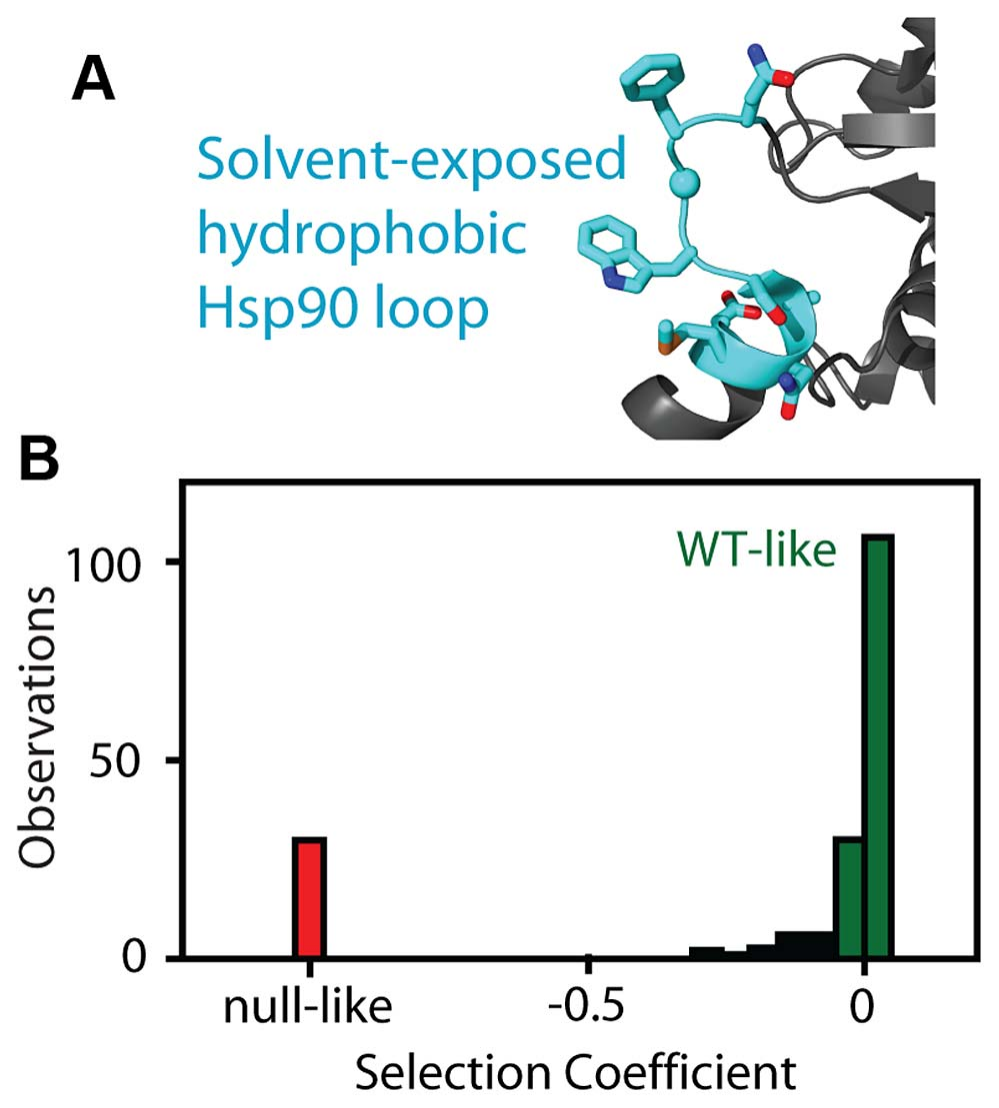
Fitness effects of Hsp90 amino acid substitutions. (A) The fitness effects of all possible amino acid substitutions were analyzed for the region highlighted in cyan. This representation is based on the crystal structure of yeast Hsp90 [50]. (B) At endogenous expression strength, the distribution of fitness effects was bimodal with many mutations resulting in either WT-like (green) or null-like (red) growth rates.

We analyzed the fitness effects of Hsp90 point mutants by performing a bulk competition in the DBY288 strain. A library of plasmids containing all possible single codon substitutions at amino acid positions 582–590 (Figure 1A) was transformed into a single batch of yeast. These experiments used a plasmid and promoter construction previously shown to match the endogenous expression level of Hsp90 [42]. Transformed yeast cells were preferentially amplified in galactose media that allowed all mutations including null alleles to propagate. The bulk culture was transferred to shutoff conditions to initiate selection on the mutant library. The beginning of strong selection on the mutant library was estimated from the growth plateau of control cells harboring a null rescue plasmid (Supplementary Figure S1). After the initiation of selection on the mutant libraries, samples were harvested over the following 36 hours and the relative abundance of each mutant quantified using focused deep sequencing. By comparing the trajectory of each mutant relative to wild type, we directly determined competitive advantage or disadvantage of each amino-acid substitution as an effective selection coefficient (s) that represents the competitive asexual growth advantage/disadvantage of each mutant in a defined environment [29]. We have previously demonstrated that the EMPIRIC approach provides highly reproducible measures of fitness effects that strongly correlates with the growth rate of individual mutants grown in monoculture [43]. Consistent with our previous work, effective selection coefficients were highly reproducible (R^2^ = 0.96) in a full experimental repeat (Figure S2). At the endogenous expression strength, the distribution of fitness effects for this region of Hsp90 was bi-modal (Figure 1B, Supplementary Table S1), with peaks near wild type and null. Bi-modal fitness distributions are predicted based on a model where fitness effects are dominated by the impact of mutations on protein stability [18]. Thus, our fitness analyses at wild type expression level are consistent with this region of Hsp90 serving a primarily scaffolding purpose.

**Figure 2:**
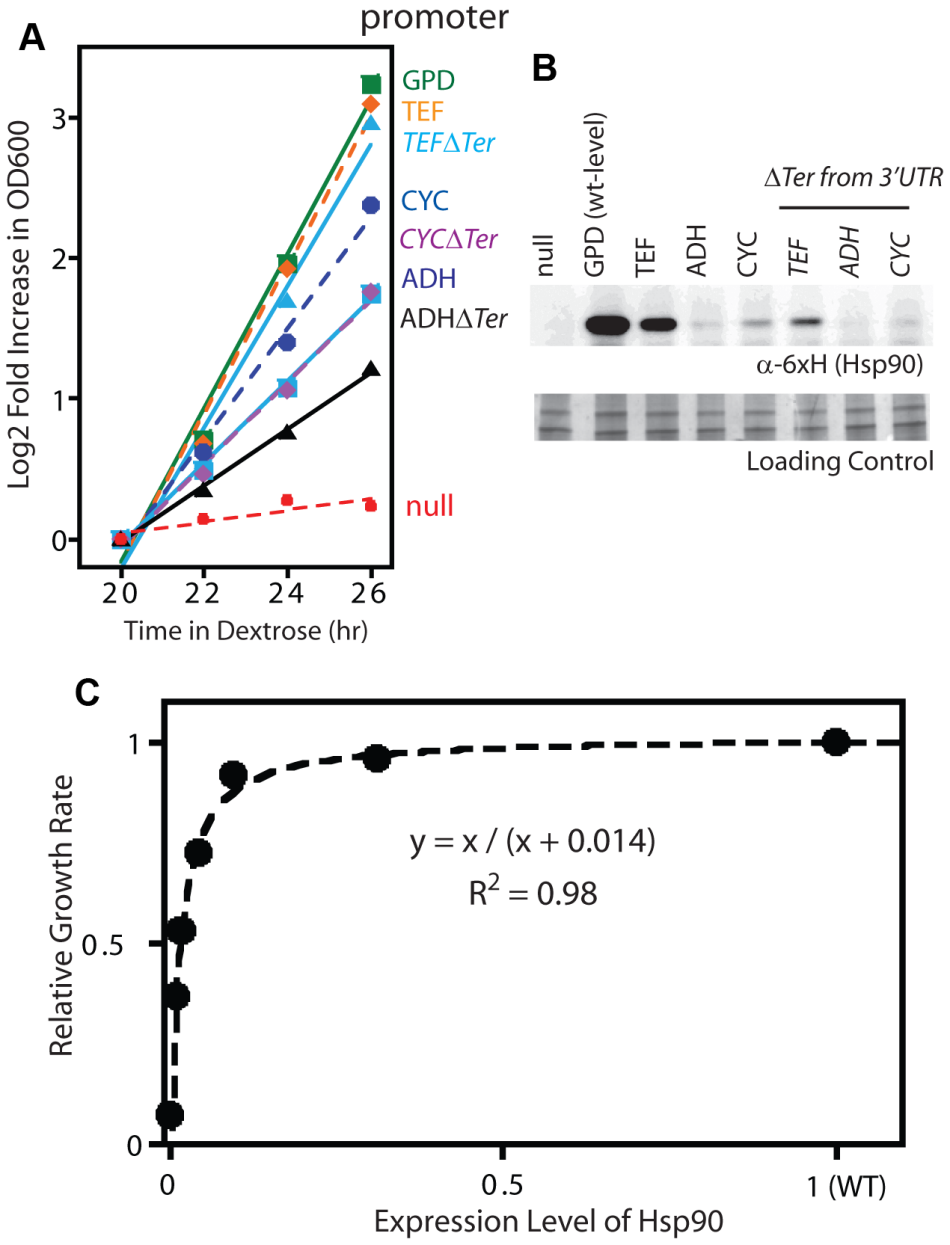
Effect of reduced Hsp90 expression on yeast growth. (A) Growth of Hsp90 shutoff yeast harboring rescue Hsp90 plasmids with varied promoters with or without a terminator in the 3′ UTR. Yeast were grown at 30°C and monitored by optical density at 600 nm. (B) Hsp90 expression in cells grown in dextrose for 19 hours was monitored by Western blotting. (C) Relationship between observed Hsp90 expression level and growth rate.

To further probe the relationship between the net function of Hsp90 and fitness, we varied expression level of the WT sequence and analyzed impacts on growth rate (Figure 2). To vary expression level, we swapped both promoter and terminator (3′ untranslated) sequences. Closely following the start of strong shutoff selection (19 hours in dextrose), we observed a 2-fold range in growth rate with these constructs (Figure 2A) and a 100-fold range in expression level (Figure 2B). We quantified expression level using a Western blot assay directed against an 6×His epitope tag only present on the rescue copy of Hsp90 that we had previously optimized to yield a linear response [44]. These expression level measurements were performed after 19 hours in dextrose, where the second copy of Hsp90 driven by the galactose regulated promoter was undetectable (Supplementary Figure S1). To further investigate expression level, we developed an Hsp90-GFP fusion construct that we monitored by flow cytometry. Across all promoter constructs, the Hsp90-GFP fusion supported similar yeast growth rates to non-GFP tagged versions (Supplementary Figure S3). These findings indicate that the GFP fusion has minimal impacts on Hsp90 function. The expression levels determined by GFP and flow cytometry were in close agreement with those measured by Western blotting and the average of both measures was used to estimate expression levels (Supplementary Table S1).

Both the Western and GFP experiments demonstrate that the expression level of Hsp90 can be reduced dramatically (15-fold) without major impacts on growth rate, which is consistent with previous reports [22], [45]. The growth rate to Hsp90 expression level profile that we determined has the shape of a binding curve (Figure 2C), and can be fit to a binding equation that represents the elasticity function for Hsp90. This elasticity function defines how yeast growth rate varies with the net Hsp90 function and enabled us to calculate per molecule function of mutants from fitness measurements.

**Figure 3:**
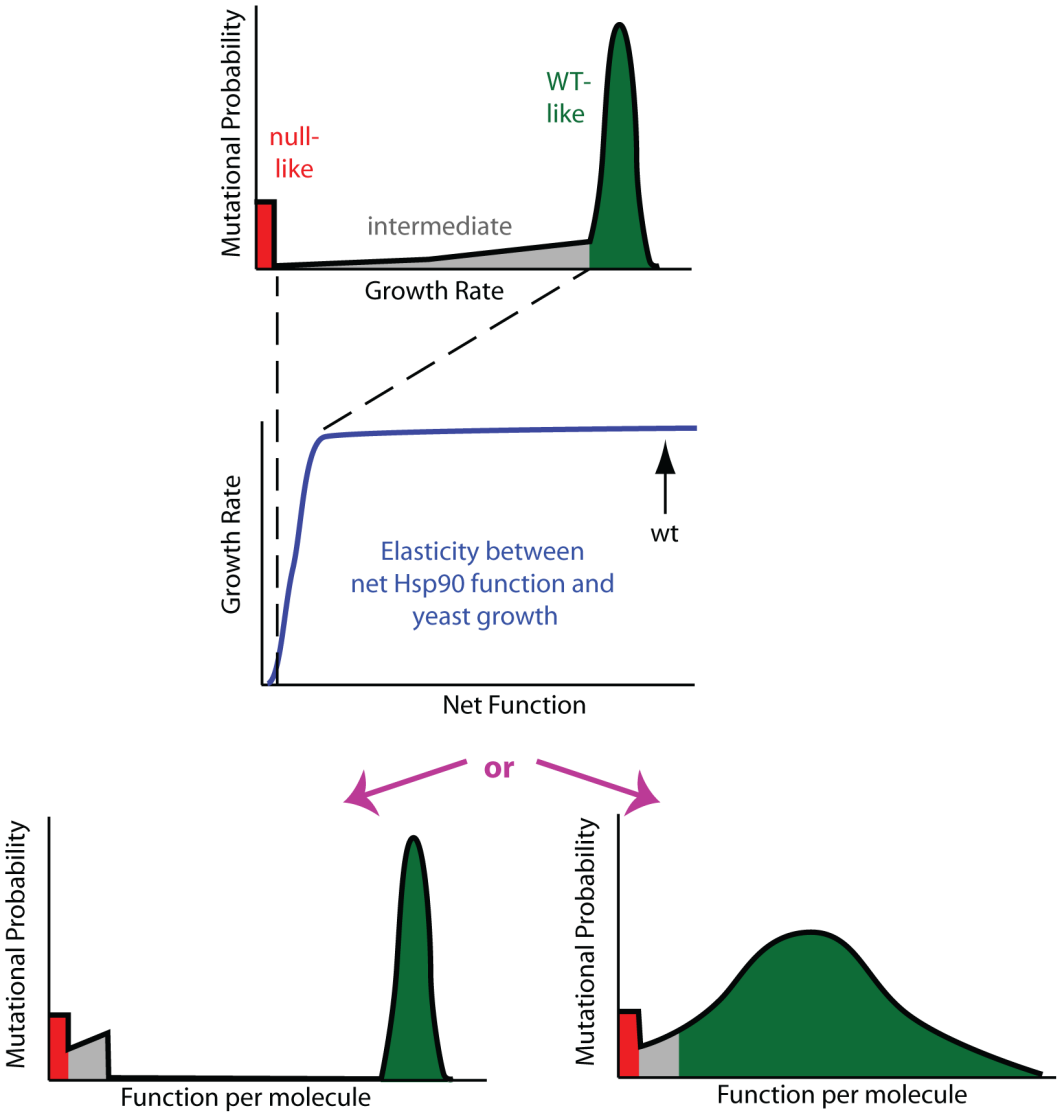
Mutant effects on protein function can be hidden to fitness analyses. Fitness effects (top panel) are a property of the elasticity relationship between net protein function and growth (middle panel) and the impact of mutations on function per molecule (bottom panels). For Hsp90, the non-linear elasticity relationship could mask defects, making multiple distributions of functional effects (bottom panels) indistinguishable to fitness analyses. In the top and bottom panels, green represents mutants with WT-like, red null-like, and grey intermediate fitness effects.

The non-linear elasticity function for Hsp90 describes the coupling of mutant effects on function and fitness. For example, when expressed at endogenous levels, an Hsp90 amino acid substitution would need to reduce per molecule function by 79% in order to result in a readily measureable growth defect of 5%. Thus the bimodal DFE that we observe for Hsp90 (Figure 1B) does not necessarily imply a bimodal distribution of mutant effects on function. In particular, the fitness analyses do not provide detailed information on mutants with up to 79% defects in function. Due to the shape of the Hsp90 elasticity curve, the bimodal DFE is consistent with either a bimodal distribution of function as predicted by the stability dominated fitness model [18], or a primarily unimodal distribution of functional effects (Figure 3). To distinguish between these possibilities we sought to reveal effects on function that could be hidden at wild type expression strength.

**Figure 4:**
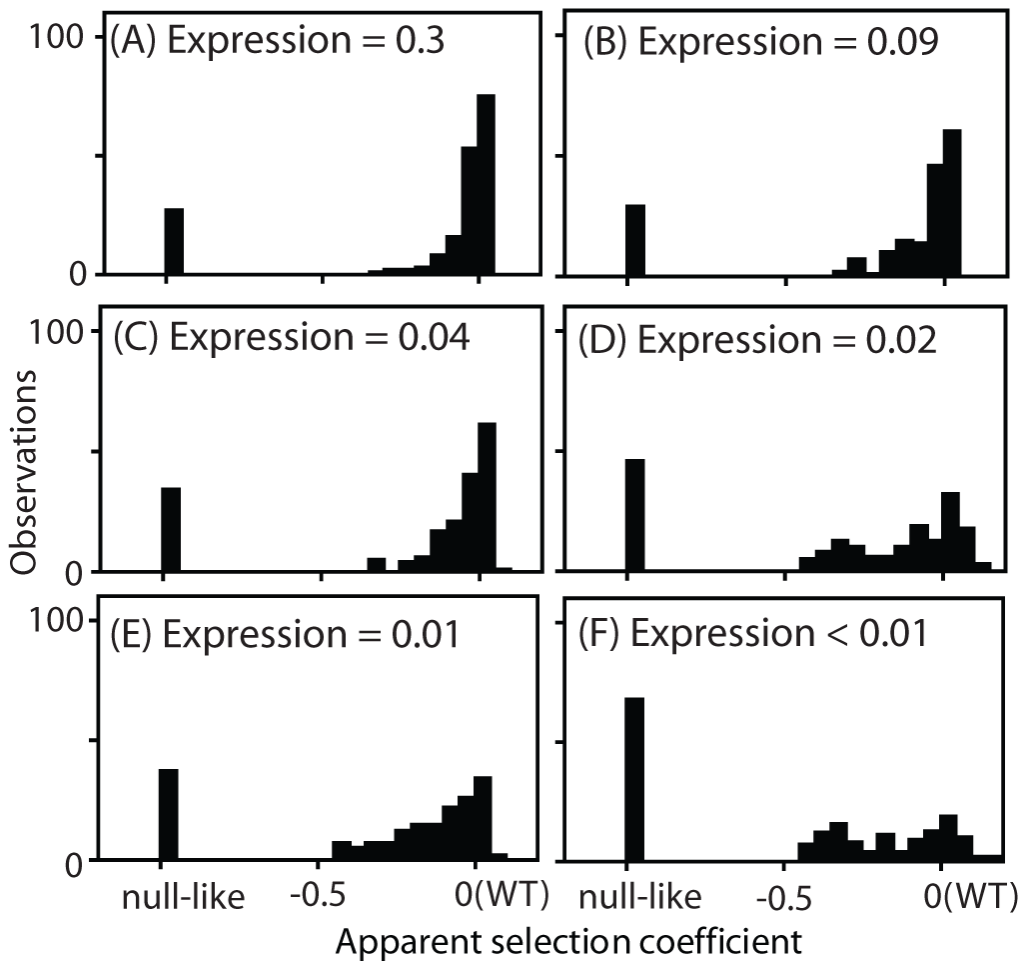
Distribution of observed fitness effects. EMPIRIC results for Hsp90 mutants with varied promoters with and without terminator sequences in the 3′ UTR: (A) TEF promoter, (B) TEF promoter without a terminator, (C) CYC promoter, (D) CYC promoter without a terminator, (E) ADH promoter, and (F) ADH promoter without a terminator.

To reveal the latent function of Hsp90 mutants, we analyze fitness effects at reduced expression strengths (Figure 4, Supplementary Table S2). The population in all bulk competitions was managed such that the population size at constriction points was always in gross excess to library diversity (Supplementary Figure S4). Because there is selection pressure to increase expression in these experiments, we examined the expression level of the wild type Hsp90 sequence over time in shutoff conditions using Hsp90-GFP fusions (Supplementary Figure S5). Cells respond to selection by increasing expression from weak promoters over time. As predicted by the elasticity function (Figure 2), the increased expression from weak promoters results in an increase in growth rate (Supplementary Figure S6). The observed increase in growth rate closely matches predictions based on the expression increase we observed by flow cytometry and the elasticity function, indicating that the underlying model is sound. To minimize the impact of time dependent changes in expression on fitness analyses of coding sequence mutations, we performed bulk competition of Hsp90 mutants over a short time window, 12–48 hours in dextrose (Supplementary Figure S4). We performed simulations to investigate how the observed increase in expression level over time in shutoff conditions would impact competition trajectories (Supplementary Figure S7). The impact of increasing expression level has a minor impact on competition trajectories and indicates that constant expression models provide estimates of sufficient quality to interpret general features of the distribution of mutant effects on fitness and function, which is the focus of this study.

The DFEs that we observed exhibited a consistent trend as expression strength was reduced. At high expression strength, the majority of mutants had WT-like growth rates, with very few mutants of intermediate effect. As expression strength was reduced, the WT-like peak decreased and the prevalence of mutants with intermediate effects increased. In terms of epistasis, the fitness effects of amino acid substitutions displayed pervasive negative epistasis with expression strength (Supplementary Figure S8). In terms of function, these results strongly indicate that the DFE at endogenous expression strength (Figure 1B) does not mirror the underlying effects of point mutations on Hsp90 function.

**Figure 5:**
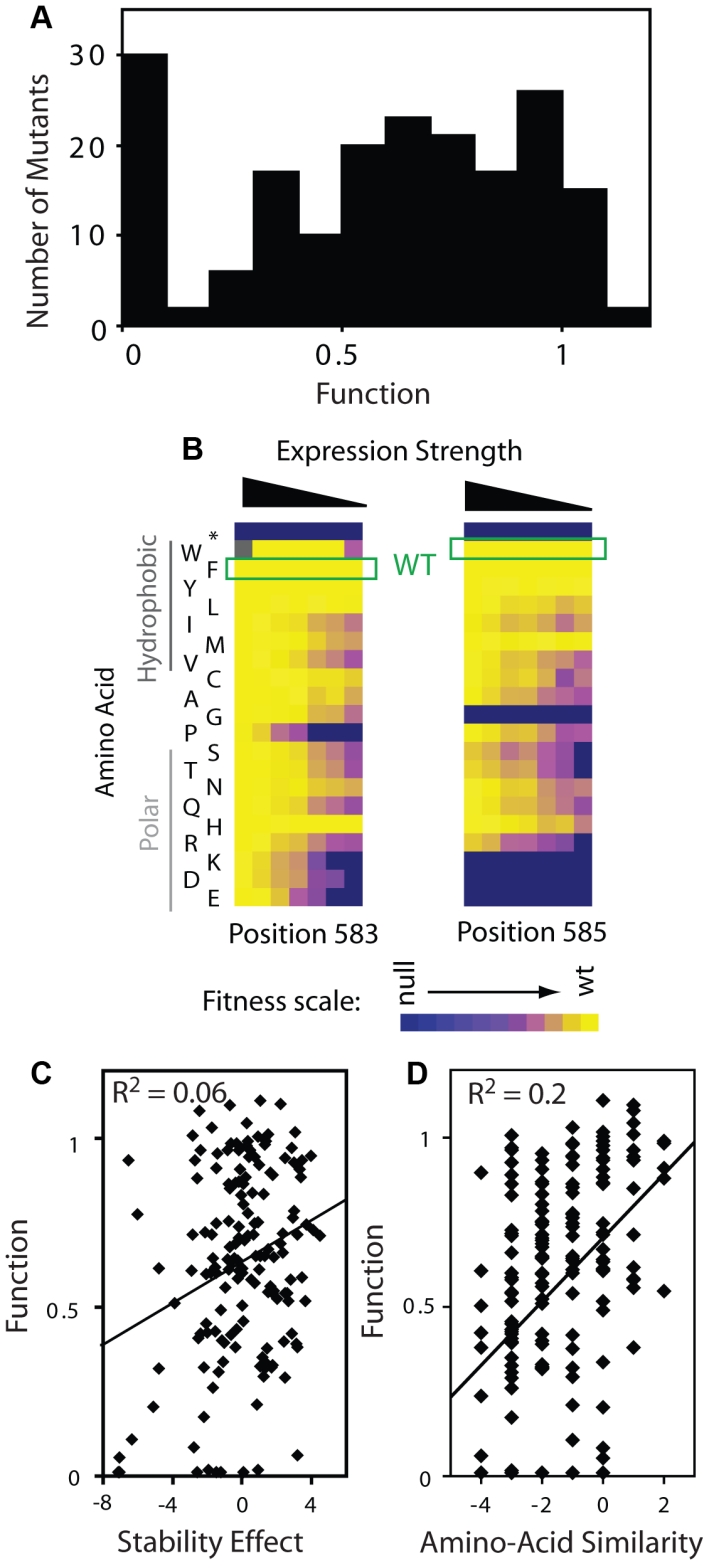
Effects of mutations on Hsp90 function. (A) Distribution of mutant effects on Hsp90 function calculated from fitness effects at varied expression levels. (B) Impact of mutations at two solvent exposed hydrophobic amino acids in Hsp90 on yeast growth at different expression strengths. (C) Mutant impacts on folding stability (−ΔΔG estimated from structural simulation) related to function. (D) Similarity of amino acid substitutions to wild type (based on Blosum62 matrix) relative to observed functional effects.

We estimated mutant effects on Hsp90 function (Figure 5, Supplementary Table S3) based on fitness measurements at distinct expression strengths and the elasticity function. As described in the methods section, we employed the elasticity function to calculate per molecule function from fitness taking into account bounds on measurement and calculation precision. For example, at the endogenous expression strength, mutants with activity defects of up to 79% were obscured to fitness analyses and were demarcated as such (functional efficiency >0.21). Because a distinct range of function is revealed to selection at each expression strength (Table 1), our integrated analyses provided estimates of the functional effects of all mutants. Estimates of mutant effects on function based on fitness measurements at different expression strengths exhibit a reasonable correlation (R^2^ = 0.75) (Supplementary Figure S9). The strength of this correlation, despite simplifying assumptions (further discussed in the methods section), indicates that the calculated mutant effects on function are fair estimates.

**Table 1:** Activity ranges interrogated at each expression-strength.

Construct Name	Expression Strength	Function Range^1^
GPD	1.0	0.034–0.21
TEF	0.32	0.067–0.45
TEFΔ*Ter*	0.094	0.16–0.71
CYC	0.044	0.38 and above
CYCΔ*Ter*	0.019	0.38 and above
ADH	0.014	0.39 and above
ADHΔ*Ter*	0.008	0.43 and above

1 Function range with informative fitness effects (s_app_>null-like – where mutants persist in the culture and are more accurately monitored; and observed growth rates at least 5% slower than G_max_ where growth rate and function are strongly coupled.

The distribution of functional effects for a region of a protein provides information about the contributions of that region to biochemical activity. For example, scaffolding regions that are not directly involved in a critical or rate-limiting step in protein function should be hard to break by mutation (due to selection for stability in the wild type protein), but once broken destroy activity [19], [20]. In contrast, regions that contribute to a rate-limiting step should be easy to injure by mutation, with the severity of mutant defects mediated by the rigidness of chemical and physical requirements (e.g. catalytic sites in enzymes being ultimately rigid with any mutation destroying activity).

The distribution of functional effects (Figure 5A) for the region of Hsp90 we analyzed had one main peak with most mutations exhibiting partial defects relative to wild type. Our finding is consistent with this region of Hsp90 contributing to a critical and rate-limiting step in function. The intermediate functional defect of most mutants indicates that the chemical and physical requirements are flexible, consistent with this region of Hsp90 providing a hydrophobic docking site for binding to substrates, as was inferred from structure [32]. Taking a closer look at the aromatic amino acids at position 583 (Phe) and 585 (Trp) located on the surface of the Hsp90 structure, most amino acid substitutions are tolerated when expressed at endogenous levels, but a clear functional preference for hydrophobic amino acids is revealed at reduced expression strengths (Figure 5B). Hydrophobic interactions [46] are malleable to slight alterations in geometry and physical composition compared to other physical interactions (e.g. hydrogen bonds). Thus, it is reasonable that some substitutions that maintain hydrophobicity would be well tolerated, but that most non-conservative substitutions would result in strong defects.

Our fitness-based estimates of mutant effects on function integrate over all properties that contribute to cell growth including catalysis, binding affinity, as well as the thermodynamic stability of folding to the native state [18]–[20], [27], [47]. In terms of stability, the prevalence of intermediate functional defects that we observe is inconsistent with this region of Hsp90 serving a purely scaffolding function, which theory predicts should exhibit a bi-modal distribution [18]. Furthermore, we observed a similar distribution of functional effects for positions located on the protein surface, which should have relatively small impacts on stability [48], as those that orient towards the protein interior (Supplementary Figure S9). This finding suggests that the functional effects of mutants at solvent shielded positions are caused primarily by local structural changes that impact the organization of solvent exposed positions (e.g. as required for efficient binding to substrate). We have observed a similar surface-core relationship in ubiquitin [43], and at a lower resolution this type of surface-core association has been postulated based on the slow evolutionary divergence of sites in proteins located proximal to binding sites [49]. Of note, Hsp90 is a dimeric protein and subunit folding and association are coupled [44]. Thus, decreased expression strength could increase sensitivity to destabilizing mutations. In this case, destabilizing mutations would exhibit larger activity defects at lower expression strength. Across the dataset our functional estimates are largely independent of expression strength (Supplementary Figure S9, Panel A). Thus, the effects of mutations on dimer stability appear to have at most a minor impact on our activity estimates, consistent with the location of this region of Hsp90 far from the dimer interface [50].

To further examine the effect of mutations on stability, we simulated the stability effects of each possible point mutation based on the structure of Hsp90 [50] using Rosetta [51], which accurately predicts the experimental effects of mutations on stability. The simulated stability effects for Hsp90 correlate extremely weakly with activity (Figure 5C), consistent with our conclusion that stability is not a dominant contributor to activity for this region of Hsp90. Of note, substitutions of amino acids with similar physical and chemical properties (as estimated by BLOSUM similarity) to the wild type residue tend to be compatible with function (Figure 5D). The stronger correlation of function with amino acid similarity compared to stability suggests that the stability simulations do not fully capture all biologically relevant structures. For example, high resolution structures of Hsp90 bound to substrate are not available; but if they were available, might provide a stronger structural explanation for the observed functional effects of mutations.

**Figure 6:**
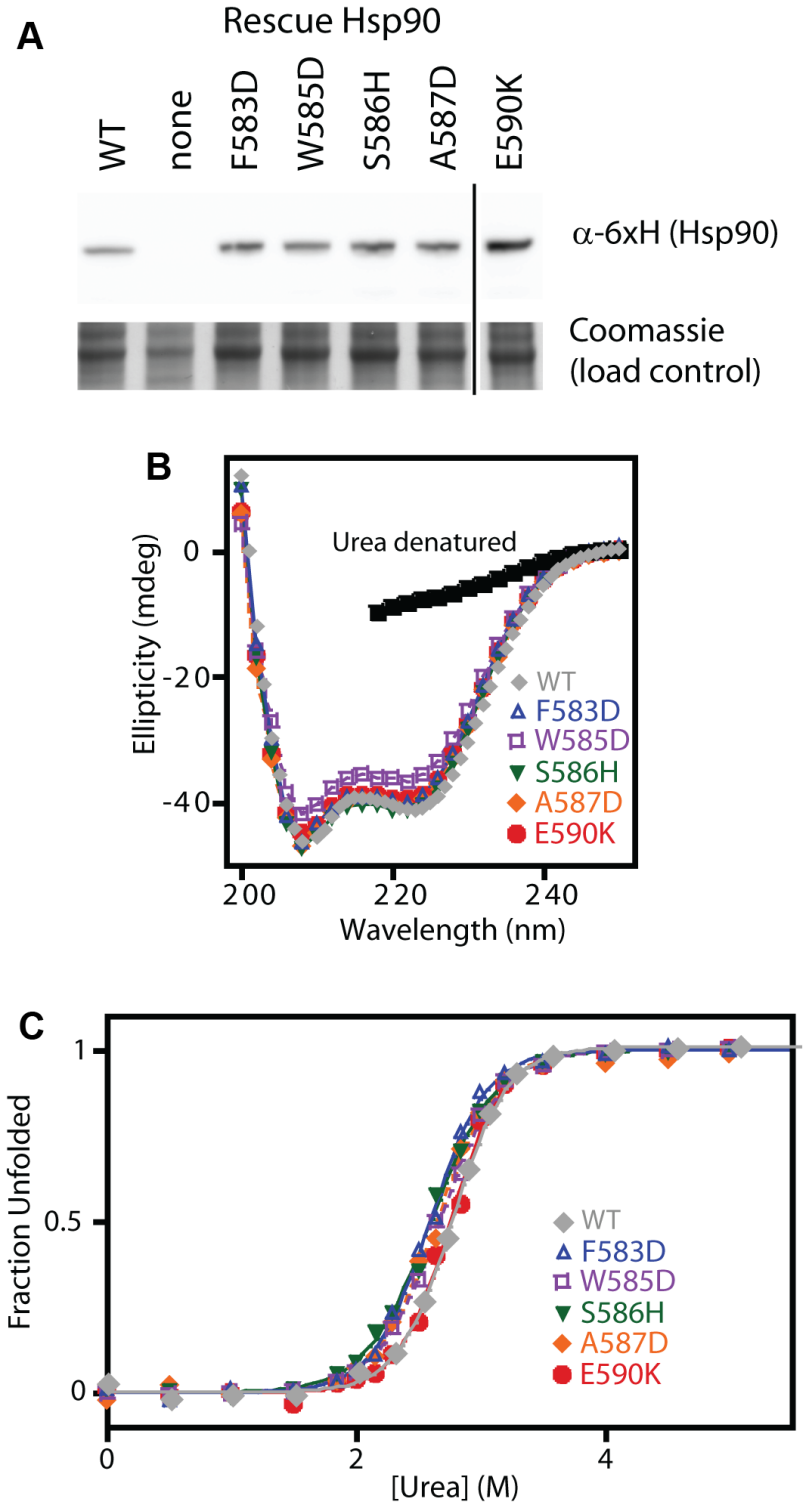
Expression level and stability of five non-conservative mutations. (A) Expression level in shutoff yeast analyzed by Western blotting. The vertical line represents intervening lanes that were removed for clarity. (B) Secondary structure of purified C-domain constructs analyzed by circular dichroism. The spectrum of a denatured sample in 5M urea is shown for comparison (below 218 nm absorbance from urea interferes with signal). (C) Urea induced unfolding of purified C-domain constructs. The fraction unfolded was determined based on ellipticity at 222 nm.

To further test our model and conclusions, we experimentally investigated the biochemical properties of five non-conservative amino acid substitutions. We chose mutations that dramatically change the hydrophobic binding surface and largely destroy function (F583D and W585D), mutations that disrupt intra-molecular interactions and severely impair function (S586H disrupts a buried hydrogen bond, and A587D introduces a buried charge at a solvent shielded location), and a charge reversal mutation (E590K) on the surface that causes a moderate functional defect. The growth rate of these mutants in monoculture closely matched the fitness effects observed in the bulk competitions (Supplementary Figure S10). As discussed above, our estimates of function integrates over multiple protein properties. For example, a mutation that increases the degradation rate (with the synthesis rate unchanged) should exhibit reduced steady state levels leading to a defect in net function. All of the disruptive individual mutations that we investigated accumulated at similar steady state levels (Figure 6A), suggesting that individual mutations do not commonly disrupt Hsp90 protein levels.

We examined the biophysical properties of these non-conservative Hsp90 mutant proteins in purified form. To maximize the sensitivity of these analyses for potential alterations to structure and stability, we generated C-domain constructs. All of the mutations we analyzed are located in the C-domain and do not contact other domains in the Hsp90 structure. The circular dichroism (CD) spectra of all five mutant proteins overlay closely with WT (Figure 6B) indicating that all of the mutants fold into native conformations with similar secondary structure content to WT. We investigated the stability of each mutant protein to urea-induced unfolding (Figure 6C). Similar concentrations of urea were required to unfold all mutants and WT indicating that none of the mutants compromises folding under native conditions. These findings demonstrate that non-conservative mutations in this region of Hsp90 are generally capable of folding to stable native states, and strengthen our conclusions that the 582–590 region of Hsp90 that we analyzed is not critical for folding stability, and is instead a structurally malleable region that forms a critical hydrophobic docking site.

Our studies as well as those of others [21], [24], [25], [27], [52], [53] demonstrate that biochemical flux models and the elasticity function in particular provide a fundamental link between molecular and cellular/organismal properties. Non-linear elasticity functions of the identical form to those described here for Hsp90 have also been observed in *E. coli* for β-galactosidase[53], isopropylmalate dehydrogenase [24], and dihydrofolate reductase (DHFR) [25]. In *E. coli*, DHFR point mutations were commonly observed to impact protein degradation rates leading to fitness effects that were strongly dependent on the level of protein quality control [25]. In addition, flux models can provide a mechanistic explanation for many common fitness features including pleiotropy and epistasis [54].

This study clearly demonstrates that functional defects of mutants can be hidden to experimental fitness measurements due to a non-linear elasticity function. Uncovering these latent effects revealed that the region of Hsp90 we analyzed contributes to a rate-limiting step in Hsp90 function. These findings indicate that critical functional regions in proteins are more prevalent than considered based on fitness analyses performed without consideration of the elasticity function. The elasticity function relating net function and fitness is critical for a thorough understanding of mutant fitness effects.

## Methods

### Plasmid and strain construction

For expression analysis, the yeast Hsp90 gene was cloned into the pRS414 plasmid with different promoters and 3′ untranslated region (UTR). We used constitutive promoters previously demonstrated to generate a wide variation in expression level [55] including GPD, TEF, ADH, and CYC. Constructs were generated with or without the 3′ UTR from the CYC gene, which allowed further variation in expression level [56]. In constructs lacking the CYC terminator, the 3′UTR was composed of sequence from the plasmid vector. All Hsp90 plasmids contained a 6X-His sequence (GGHHHHHHGGH) at the N-terminus to facilitate detection by Western blotting. Point mutant libraries previously generated in p417 plasmids [9] were transferred to the pRS414 promoter variant plasmids using SLIC cloning [57]. Briefly, for each promoter strength construct, we prepared a destination vector with the first and last 30 bases of Hsp90 bracketing a unique SphI restriction site. We excised the Hsp90 library from the original 417GPD plasmid using restriction enzymes that cut immediately upstream and downstream of the Hsp90 gene. We cut destination vectors with SphI. We generated ∼30 base complementary overhangs using T4 DNA polymerase in both the destination vectors and the Hsp90 library, annealed the complementary DNA, transformed into competent bacteria, grew in bulk selective (Amp) cultures and prepared plasmid. A small portion of the transformation was plated and the number of independent transformants (∼30,000) was in gross excess to the library diversity. In addition, all replication is performed in bacteria where multiple systems ensure high fidelity reducing the probability of undesired secondary mutations. The DBY288 Hsp90 shutoff strain (can1-100 ade2-1 his3-11,15 leu2-3,12 trp1-1 ura3-1 hsp82::leu2 hsc82::leu2 ho::pgals-hsp82-his3) was generated from the Ecu Hsp90 plasmid swap strain [42] by integration of Hsp90 driven by a GalS [41] promoter together with a HIS3 marker into the HO genomic locus.

### Yeast growth rate

DBY288 cells were transformed with pRS414 plasmids and selected on synthetic raffinose and galactose (SRGal) plates lacking tryptophan (-W). Single colonies were then grown in liquid SRGal-W on a rotator at 30°C to late-log phase (OD_600_∼0.8). Cells were collected by centrifugation, washed with synthetic dextrose (SD) –W media, and then grown in SD-W medium at 30°C in an orbital shaker. Culture density was maintained in log phase (OD_600_ between 0.1 and 0.8) by periodic dilution. Culture growth was monitored based on increases in OD_600_ taking into account cumulative dilution. The log of OD_600_ versus time was fit to a linear equation to determine growth rate. Analyses were performed on time points in dextrose where control cells lacking a rescue Hsp90 had depleted Hsp90 by Western analyses (Figure 2B & Supplementary Figure S1) and had stalled in growth (Figure 2A and Supplementary Figure S1).

### Analyses of Hsp90 expression level by Western

To analyze expression levels of different promoter constructs, cells were grown for 19 hours in SD -W media, and 10^8^ yeast cells were collected by centrifugation, and frozen as pellets at −80°C. Cell lysates were prepared by vortexing thawed pellets with glass beads in lysis buffer (50 mM Tris-HCl pH 7.5, 5 mM EDTA and 10 mM PMSF), followed by addition of SDS to 2%. Lysed cells were centrifuged at 18,000 *g* for 1 minute to remove debris, and the protein concentration of the supernatants was determined using a BCA assay (Pierce Inc.). Lysates with 15 µg of cell protein were resolved by SDS-PAGE, transferred to a PVDF membrane, and Hsp90 probed using α-HisG antibody (Invitrogen Inc.). Importantly, we have previously shown that detection of this 6×His Hsp90 construct in yeast can be detected with a broad linear range using this antibody and Western blot approach [44].

### Analyses of Hsp90 expression level using flow cytometry

Flow cytometry was used as an alternative approach to measure the expression level of Hsp90 at the single cell level in yeast cells. A gene encoding EGFP was inserted into the unstructured tail of Hsp90 after amino acid position 684. This Hsp90-GFP fusion construct was cloned into the variable strength promoter constructs used with non-GFP tagged Hsp90. These plasmids were transformed into DBY288 yeast competent cells and grown on SRGal-W plates. A single colony of each strain was grown for two days at 30°C in SRGal-W media to near saturation. These cultures were diluted 1∶50 into SRGal-W media and grown to late log phase (∼10^6^ cells/ml). Each strain was then further diluted 1∶50 in SD-W media for 48 hours at 30°C with dilution every 12 hrs in order to maintain cells in log phase growth. Samples of cells were collected after 19, 36, and 48 hours in dextrose. Collected cells were washed twice in wash buffer (50 mMTris, 150 mMNaCl, pH 7.6, 0.1% w/v BSA), diluted to 10^7^cells/ml in wash buffer and analyzed on a Becton-Dickinson FACSCalibur flow cytometer equipped with a 15 mW air cooled 488 nm argon-ion laser using a 530 nm high-pass filter. Greater than 100,000 cells were analyzed for each sample. Data were processed and analyzed using FlowJo software. Debris including clumped cells was excluded by gating on the forward and side scatter (excluded less than 5% of points). To compare with bulk Western measurements, mean fluorescence was calculated using cells without GFP in order to subtract out background due to autofluorescence.

### Circular dichroism

C-domain constructs of Hsp90 bearing an N-terminal 6×His tag were generated in a bacterial over-expression plasmid, expressed, purified, and analyzed by circular dichroism (CD) as previously described [44]. Briefly, CD spectra were obtained using a 1 mm path length cuvette at a protein concentration of 20 µM in 20 mM potassium phosphate at pH 7 and 25°C. Urea titrations were performed under the same conditions using samples that were equilibrated for 30 minutes. Urea concentrations were determined based on their refractive index. CD ellipticity at 222 nm was used to follow urea induced unfolding and the resulting data was fit to a two-state unfolding model as previously described [44].

### EMPIRIC analyses of point-mutants

The effect of point mutants on yeast growth was analyzed as previously described [36]. Time points in dextrose were selected for analysis where control cells lacking a rescue Hsp90 began to stall in growth in order to observe the rapid decrease in relative abundance of deleterious mutants (e.g. premature stop codons). The growth rate of cells harboring the WT coding sequence in bulk competitions was estimated from monoculture growth of WT constructs performed in parallel to the bulk competitions. For the GPD, TEF and TEFΔter constructs we analyzed time points in dextrose of 12, 16, 20, 24, 32, 40, and 48 hours (Supplementary Table S4). For the CYC, ADH, CYCΔter, and ADHΔter constructs where the relative decrease of deleterious mutants was less severe (due to slower growth rate of fit mutants) we analyzed time points in dextrose of 16, 20, 24, 32, 40, and 48 hours. To process these time point samples, yeast pellets were lysed with zymolyase and total DNA was extracted and purified through a silica column. The DNA encoding amino acids 582–590 was PCR amplified, and prepared for 36 base single-read Illumina sequencing. 3.4×10^7^ high quality reads (>99% confidence across all 36 bases) were obtained and analyzed. The relative abundance of each point mutant at each time point for each promoter was tabulated. Effective selection coefficients for yeast growth were determined by linear fits to the change in mutant abundance relative to wild type for each possible codon substitution. To account for the rapid depletion of null-like mutants to noise levels, only the first three timepoints in selection were used to determine effective selection coefficients for stop codons and all other mutants with effective selection coefficients within two standard deviations of stop codons (corresponding to *s* = −0.28 for GPD, *s* = −0.37 for TEF, *s* = −0.4 for TEFΔter, *s* = −.0.35 for CYC, *s* = −0.46 for ADH, *s* = 0.44 for CYCΔter, and *s* = −0.43 for ADHΔter). Because these null and near-null mutants rapidly deplete from the culture it is challenging to precisely measure their relative growth effects and they were binned as “null-like” (Supplementary Table S2).Potential noise was analyzed by calculating normalized residuals (residuals/time points fit). Codon substitutions with residuals per time point greater than 0.25 or low initial mutant abundance (mutant/wt less than 0.004) were omitted (∼7% of codons). For mutants that persist in the bulk competition (*s*>−0.1) synonymous codons exhibit a narrow distribution (Supplemental Figure S11) indicating that the amino acid sequence is a dominant determinant of fitness. The effective selection coefficient for each amino acid substitution was estimated as the average of the effective selection coefficients of all synonymous codons. Epistasis between expression strength and amino acid substitutions was calculated as the difference in effective selection coefficient at reduced expression strengths relative to endogenous strength. For the epistasis calculations, null-like mutants were considered as true nulls. Thus, a mutant with wild type fitness at endogenous expression strength, and null-like fitness at the reduced expression strength would have an epistasis of −1.

### Estimations of mutant effects on function

Function per molecule was calculated based on observed selection coefficients, the elasticity function, and the expression level for each different promoter construct using the following equations.
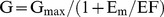
(1)

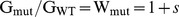
(2)


Where G is growth rate, G_max_ is the maximal growth rate, E_m_ is the relative expression level that results in half maximal growth, E is the expression level relative to the endogenous level, F is the per molecule functionof a mutant relative to WT, W_mut_ is the growth rate of a mutant relative to WT, and *s* is the effective selection coefficient. Equation 1 is an extension of the elasticity equation (Figure 2), where the expression of functional molecules or net function (EF) is explicitly modeled. With the WT coding sequence (F = 1 by definition), equation 1 simplifies to the elasticity function in Figure 2. These equations can be combined and rearranged to define F as follows.
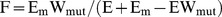
(3)


Equation 3 was used to estimate mutant effects on function (Supplementary Table S3) using the observed selection coefficients (Supplementary Table S2), E_m_ = 0.014 (Figure 2), E for each promoter construct based on experimental measurements (E_GPD_ = 1,E_TEF_ = 0.32,E_TEFΔter_ = 0.094), or estimated from the observed growth rate and the elasticity function for weak promoter constructs where experimental measures of expression were noisier (E_CYC_ = 0.028,E_CYCΔter_ = 0.015,E_ADH_ = 0.014,E_ADHΔter_ = 0.010). Where growth rates prohibited accurate estimation of fitness (null-like mutants, or absolute growth rates within 5% of G_max_), bounds on relative per molecule function were calculated (Table 1). For each amino acid substitution, a final per molecule function estimate was generated by averaging across all promoter constructs that yielded a numerical estimate (and not a bound). For all pair-wise numerical function estimates (e.g. at two different expression strengths), we compared function effects between all constructs with adjacent expression levels (Figure S9). To facilitate biophysical comparisons, we used the Blosum62 matrix [58] to calculate the amino acid similarity to wild type for each possible point mutation, and Rosetta [51] to simulate effects on thermodynamic folding stability.

### Model assumptions

We make the simplifying assumption that expression level is independent of mutations to the coding sequence. Steady state expression level is determined by the rates of both synthesis and degradation. Because degradation occurs after protein synthesis, it should depend primarily on the protein sequence such that synonymous substitutions minimally impact degradation rates. Across our data set we noted that synonymous substitutions did not have dramatic impacts on fitness, suggesting that synthesis rates were relatively independent of mutation. Protein degradation rates vary depending on protein sequence, but all of the mutants that we analyze are single amino acid substitution, and hence minimally differ in overall sequence. In the event that a point mutant impacts degradation rate, it should be consistent across each promoter construct. Thus, mutant impacts on degradation should be rare (see Figure 6), but would be incorporated into our estimates of function.

In analyzing the effect of mutations relative to wild type, we make the simplifying assumption that function is independent of expression level. We examined the validity of this assumption by analyzing the standard deviation in function for each amino acid substitution determined at different expression levels. The average standard deviation was 0.1, indicating that this assumption is valid on a rough scale (on the order of 0.1) and is appropriate for interpreting the main features of the distribution of mutant effects on function. Of note, the mutations that we observe to improve function at reduced Hsp90 expression levels (Figure 5, Supplementary Table S3) may be an artifact of this assumption.

The elasticity function does not include a cost of expression and as such has a maximum fitness at infinite expression level. Thus, we assume that expression cost is negligible relative to expression benefit over the range of our analyses. As the expression cost of native proteins is below experimental detection in yeast [59], this assumption appears reasonable.

We infer differences in cellular growth rates from measurements of DNA abundance. This inference is valid if DNA and cellular abundance are coupled. In previous work, we demonstrated that EMPIRIC measurements of fitness based on measures of plasmid abundance correlate strongly with cellular growth rates for a large set of mutants [43], indicating that plasmid abundance and cellular abundance are coupled. In addition, the copy number of the CEN plasmids utilized in this study is regulated, as cells maintaining multiple CEN plasmids grow slowly [60]. In addition, the low copy number of CEN plasmids is dominant to the addition of high copy genetic elements [61] and genetic alterations that increase CEN abundance are rare [62]. Nonetheless, CEN plasmids are not as stable as chromosomally encoded DNA, which may lead to a small amount of noise in our measurements.

## Supporting Information

Figure S1 Hsp90 shutoff strain. (A) The DBY288 strain grows robustly in dextrose when provided with a rescue plasmid that constitutively expresses Hsp90, but stalls in growth with a null-rescue plasmid. (B) Expression level of Hsp90 in DBY288 is near-endogenous in media with galactose (SRGal), but below Western blot detection after 19 hours in dextrose media (SD).
(EPS)

Figure S2 Correlation between effective selection coefficients measured in a full experimental repeat at endogenous expression level. Strongly deleterious mutants rapidly deplete in bulk competition and are not monitored as precisely as more fit mutants. Relative selection coefficients are strongly reproduced in these full experimental repeats performed on different days. The slope of this correlation is 1.7, likely due to a linear influence from estimates of the WT growth rate in these separate experiments.
(EPS)

Figure S3 Hsp90-GFP fusions. (A) Growth rate supported by Hsp90-GFP fusion in shutoff yeast. (B) Comparison of growth rates observed with and without GFP fusion. (C) GFP levels observed by flow cytometry. To estimate bulk expression comparable to the Western analyses in Figure 2, mean fluorescence was calculated and corrected for the auto fluorescence from cells lacking GFP.
(EPS)

Figure S4 Population management during bulk competitions. (A) Outline of experiment from transformation through selection. For all steps, the smallest population bottleneck is indicated. These bottlenecks were managed so that they were always in gross excess to the diversity of engineered mutations (64 codons × 9 positions = 576). (B) Population management during selective growth competition (after 12 hours in dextrose). Dashed lines represent dilutions to maintain cells in logarithmic growth, and arrows at the top indicate when samples were harvested for sequencing.
(EPS)

Figure S5 Expression of Hsp90-GFP fusions as a function of time in shutoff conditions. (A) GFP levels observed by FACS after 19 hours (grey filled), 36 hours (green dashed line), or 48 hours (red dashed lines) in dextrose for each promoter strength construct. To estimate the bulk expression level, mean fluorescence was calculated and corrected for the autofluorescence of cells lacking GFP. While the observed distribution of expression level among populations of cells was complex and will be interesting to examine in future studies, straightforward analyses of the population mean provided useful estimates for this study. (B) The fold increase relative to the 19 hour time point for each construct was plotted and fit to a linear model.
(EPS)

Figure S6 Influence of time in dextrose on growth rates. (A) Shutoff yeast harboring rescue plasmids with the WT Hsp90 coding sequence under different promoter strengths were monitored after 26 or 50 hours in dextrose. The TEF and TEFΔter constructs exhibit less than a 5% change in observed growth rate, consistent with the robust growth observed for these constructs immediately after selection begins in dextrose. (B) Relative growth rate of strains exhibiting growth defects upon selection in dextrose. Growth estimates at 50 hours were estimated from the growth rate at 26 hours, the elasticity function, and the observed increase in expression of Hsp90-GFP fusions.
(EPS)

Figure S7 Models of time-dependent changes in expression level. (A) Mathematical descriptions of numerical integration models relating abundance to function per molecule and expression level. (B) Trajectories of theoretical competitions of mutants with different impacts on function and WT. Trajectories simulations use Dt of 0.1 hours and growth and expression parameters from the CYCΔter construct (E_0_ = 0.016 - calculated from observed growth rate and elasticity function, E_m_ = 0.014 - from the elasticity function, τ = 0.11 hr^−1^ - based on flow cytometry of Hsp90-GFP fusions over time, G_max_ = 0.45 hr^−1^ - based on observed growth curves with the GPD construct). Simulations were performed for other constructs and exhibited less variation between CE and LIE models, indicating that the model is most sensitive to changes in E around E_m_. (C) Comparison of the slope of linear fits to the plots in panel B. (D) Representative data for competition trajectories of mutants in the CYCΔter constructs with linear fits.
(EPS)

Figure S8 Epistasis between expression strength and amino acid substitutions. Fitness effects of point mutants under reduced expression strength compared to endogenous expression strength. Results observed for Hsp90 mutants with varied promoters with and without terminater sequences in the 3′ UTR: (A) TEF promoter, (B) TEF promoter without a terminater, (C) CYC promoter, (D) CYC promoter without a terminater, (E) ADH promoter, and (F) ADH promoter without a terminater.
(EPS)

Figure S9 Mutant effects on function. (A) Cross-correlation between function estimates generated at different expression-strengths. Comparisons were made between non bounded estimates of function in constructs that neighbored in expression strength (e.g. GPD was compared to TEF., TEF to TEFΔter, etc.) (B&C) Functional estimates for positions oriented towards the protein interior or exterior. Distribution of mutant effects on function for positions 582, 583, and 585 that are oriented towards solvent (B), compared to positions (584, 586–590) that are oriented towards the protein core (C).
(EPS)

Figure S10 Monoculture growth of individual mutants. Individual mutants were generated in the GPD construct, introduced into DBY288 yeast, and growth observed after switching to shutoff conditions. We chose to analyze mutants with large differences in effective selection coefficients from the bulk competitions. Both mutants (W585D and A587D) with null-like EMPIRIC selection coefficients exhibited dramatic growth defects, while both mutants (E590K and F583D) with WT-like effective selection coefficients exhibited robust growth in monoculture. The S586H mutant that had an intermediate fitness defect in the bulk competitions also exhibited an intermediate growth defect in monoculture. For this data, the Spearman's rank correlation was −0.99 using identical EMPIRIC rankings for WT-like variants (WT, E590K, F583D) as well as for null-like variants (W585D and A587D). The negative sign in the correlation indicates that selection coefficients and growth are inversely related by definition.
(EPS)

Figure S11 Effects of synonymous substitutions. For all mutations that persisted in the bulk competitions (s>−0.1), we calculated synonym variance as the difference between the effective selection coefficient for each codon and the average of all synonyms encoding the same amino acid.
(EPS)

Table S1 Expression level measurements.
(DOCX)

Table S2 Apparent selection coefficients for Hsp90 amino acid substitutions under different promoter strengths.
(XLSX)

Table S3 Effects of Hsp90 mutants on function.
(XLSX)

Table S4 Sequencing counts.
(XLSX)
