# Supplementary material for: Latent Effects of Hsp90 Mutants Revealed at Reduced Expression Levels
Source: PLoS Genet. 2013 Jun 27;9(6):e1003600. doi: 10.1371/journal.pgen.1003600 (PMC3694843; doi:10.1371/journal.pgen.1003600)
Supplement: Table S1 — Expression level measurements. (DOCX) [file pgen.1003600.s012.docx]

Supplementary Table S1

*Relative Expression Measurements*

**Construct GFP/FACS Western Average**

GPD 1 1 1

TEF 0.31 0.32 0.32

TEFΔter 0.12 0.067 0.094

CYC 0.055 0.032 0.044

ADH 0.014 0.014 0.014

CYCΔter 0.026 0.013 0.019

ADHΔter 0.014 0.001 0.008
